# Recurrent Cutaneous Rosai-Dorfman Disease

**DOI:** 10.7759/cureus.6289

**Published:** 2019-12-04

**Authors:** Oren Michaeli, Mohammed Elassa, Richard Williams, Gerard Baltazar

**Affiliations:** 1 General Surgery, NYU Winthrop Hospital, Mineola, USA; 2 General Surgery, Rowan University School of Osteopathic Medicine, Stratford, USA; 3 Surgery, Carepoint Health Bayonne Medical Center, Bayonne, USA; 4 Surgery, NYU Winthrop Hospital, Mineola, USA

**Keywords:** rosai dorfman, extranodal, histiocytosis

## Abstract

Rosai-Dorfman disease (RDD) is a rare proliferative histiocytic disorder, most commonly presenting with cervical lymphadenopathy. When extranodal, a rare manifestation of the disease is the presence of cutaneous lesions. Surgical excision has shown promising results in patients with cutaneous RDD; however, no optimal management has been elucidated. We present a 60-year-old female with recurrence of left thigh cutaneous lesions consistent with extranodal RDD whose optimal management required combined used of excision and chemotherapy.

## Introduction

Rosai-Dorfman disease (RDD) is a rare proliferative histiocytic disorder first reported in 1969 by Juan Rosai and Ronald Dorfman. Most commonly, the clinical presentation of RDD is massive, painless cervical lymphadenopathy with varying symptoms dependent upon involved organ systems [1]. RDD has been reported in patients of a wide age-range with predominance among younger adults. Cutaneous RDD lesions are exceedingly rare, and recurrence of a cutaneous lesion after excision has only been reported once [2]. We present the second case of cutaneous recurrence of RDD with metastatic behavior that required optimal treatment with excision and chemotherapy.

## Case presentation

At initial presentation, the patient was a 58-year-old African American female who presented to a general surgical clinic with a left lateral proximal thigh cutaneous nodules for one-year duration. She complained of 15+ mildly tender nodules that were gradually increasing in size and unrelated to trauma or infection. Previous excisional biopsy at an outside institution had revealed RDD.

Our general surgery team carried out a wide local full-thickness excision of the left thigh tumor-involved skin and subcutaneous tissue with a split-thickness skin graft (STSG) performed in February 2017, encompassing all nodules and with grossly clear margins. 

**Figure 1:**
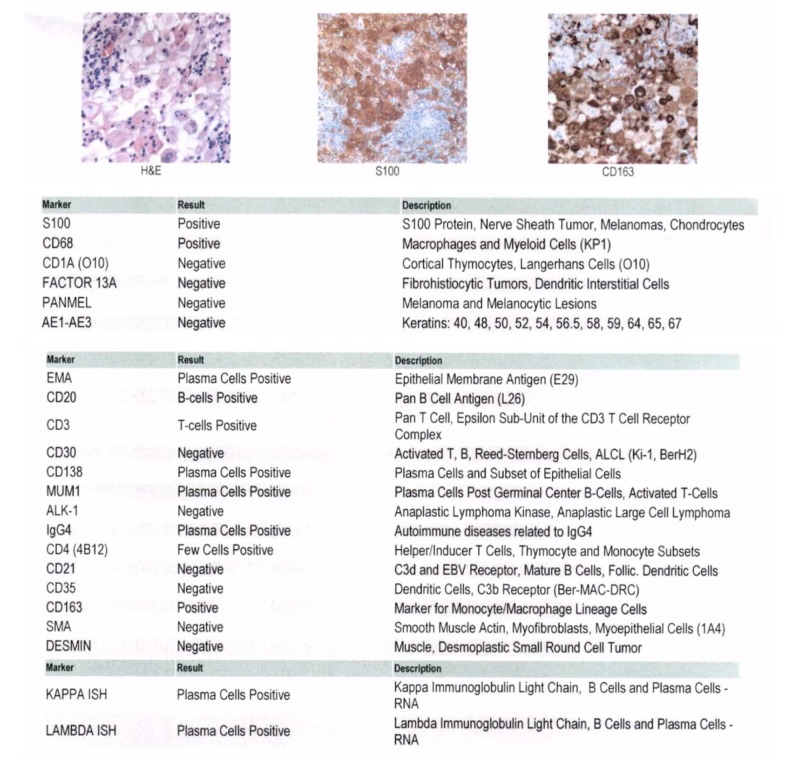
Slides provided show H&E, S100 and CD163 staining; of note, excisional samples were positive for S100/CD68, markers classically associated with RDD H&E: Hematoxylin and eosin stain; S100: Family of soluble in 100% saturated solution of ammonium sulfate; CD - Cluster of differentiation; MUM - Multiple myeloma oncogene; RDD: Rosai-Dorfman disease.

Histopathology showed predominant histiocytes and macrophages with abundant eosinophilic cytoplasm that ranged from round- to spindle-shaped. Emperiolysis was noted in histiocytes and macrophages throughout the samples. These morphological features with concomitant positive reactivity for S100 (family of soluble in 100% saturated solution of ammonium sulfate) and cluster of differentiation (CD)68 supported the diagnosis of RDD (Figure 1).

In November 2017, the patient returned to the general surgery clinic with a complaint of a nodule on the left lateral proximal thigh, proximal to the split‐thickness skin graft (STSG) in virgin tissue. Physical examination revealed non-tender, rubbery, grape-sized nodule that was freely mobile in the lateral plane and fixed to underlying tissues in the vertical plane. Wide local excision revealed diseased tissue extending and invading into the left iliotibial band. During the operation, additional lesions were palpated within the subcutaneous tissues, and dissection was continued until grossly negative margins were obtained. What was believed to be a minor lesion resulted in the excision of most of the iliotibial band.

Specimens and slides were submitted to a specialized cutaneous pathologist. The pathology demonstrated atypical mononuclear and multinuclear cell proliferation with multifocal emperipolesis most consistent with RDD with extensive areas of expansive aggregates of histiocytes positive for S100 and CD68 consistent with RDD with sarcomatous features.

**Figure 2:**
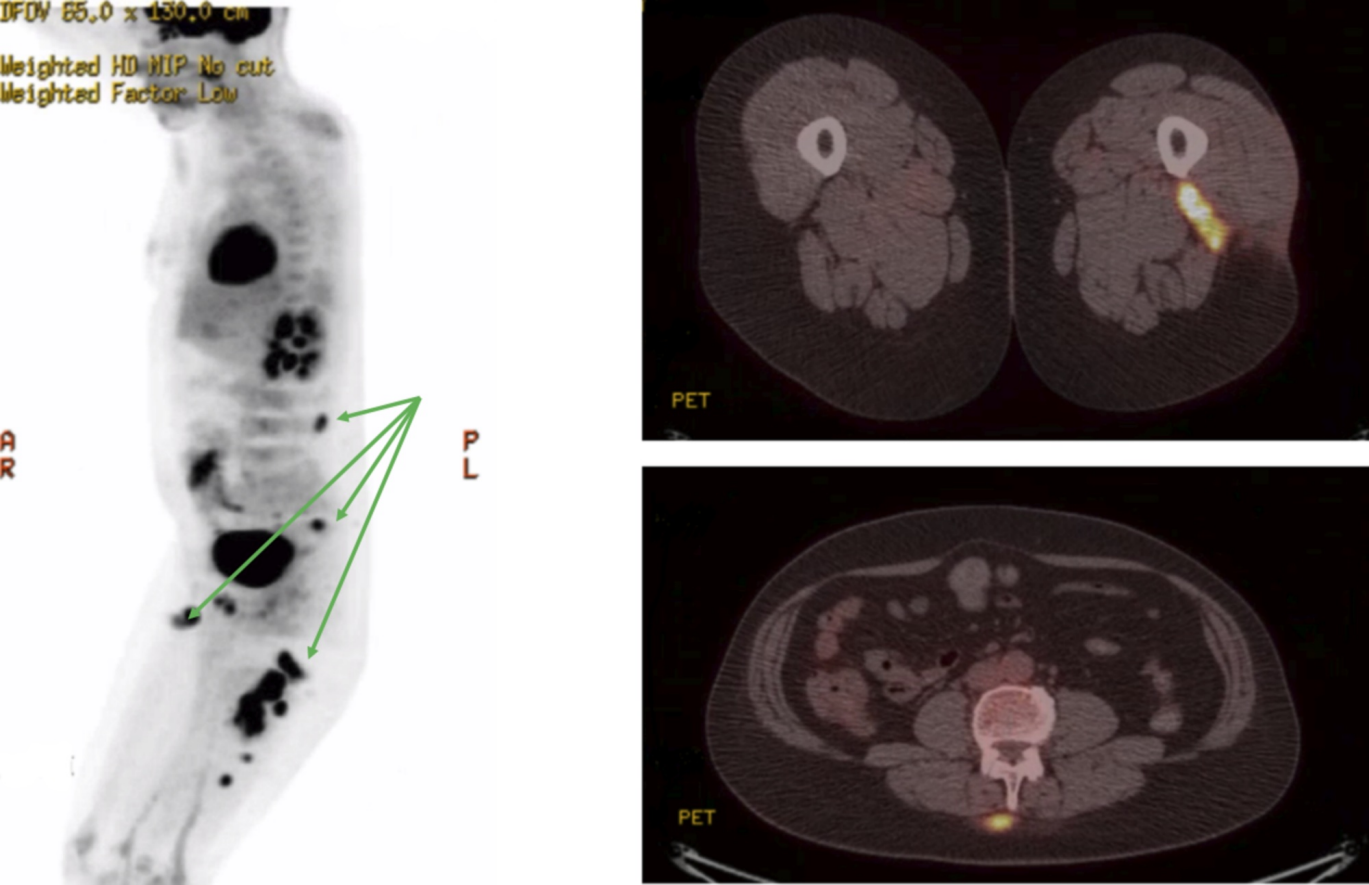
Imaging depicts multiple subcutaneous soft tissue nodules noted in the lower back at the level of L4 and in the bilateral thighs demonstrating increased fluorodeoxyglucose (FDG) uptake suggestive of cutaneous extranodal Rosai-Dorfman disease

On September 2018 as a component of follow up, a positron emission tomography/computed tomography (PET/CT) was conducted which was significant for a third recurrence this time with multiple subcutaneous soft tissue nodules noted in the lower back at the level of L4 and in bilateral thighs, demonstrating increased fluorodeoxyglucose (FDG) uptake suggestive of cutaneous extranodal RDD (Figure 2).

After the failure of multiple excisions, the multidisciplinary team overseeing her care decided to explore medical options, and the patient received intravenous cladribine (Mavenclad) daily for one week. At the first post-chemotherapy follow up with the general surgeon two months later, the palpable masses near the patient’s spine and in bilateral thighs had decreased in size to a negligible amount. Thereafter, she remained asymptomatic. 

## Discussion

RDD is identified histologically as histiocytes containing a vesicular nucleus and abundant cytoplasm with accompanying emperipolesis (i.e. lymphophagocytosis) of lymphocytes or plasma cells. The histiocytes are predominantly S100-positive and also tend to express positivity for macrophage lineage marker CD68 while the plasma cells are typically polyclonal [3].

Although most-commonly confined within the cervical lymph nodes, one study showed up to 23%-40% of patients with RDD may present with extranodal proliferation, with the most common sites of being the skin, bones, and upper respiratory tract [4]. Patients with extranodal involvement commonly present with concurrent nodal involvement as well. Purely extranodal disease is extremely rare; it has been reported to be as low as only 3% of patients of RDD [5].

The cutaneous form of the disease generally presents as deep red papules, plaques or nodules that may change in appearance over time. The cutaneous form of the disease has been reported to have a marked female predominance and a wide age distribution and without a predilection for a specific site of the body [6]. Although categorized as cutaneous, the skin lesions can infiltrate into underlying structures creating not only cosmetic complaints but also functional deficits. 

For the purposes of evaluation and staging, a widely utilized method is 18F-FDG PET/CT. Reports have shown that RDD lesions have considerable avidity for FDG as a result of their intense glucose dependence secondary to the energy requirements of proliferating histiocytes/lymphocytic inflammatory cells [5]. Imaging results are not utilized for diagnostic purposes, instead, a biopsy is required for confirmation of RDD.

Our patient benefited from follow-up PET/CT which revealed the metastatic-like behavior, prompting successful chemotherapy treatment.

Optimal treatment guidelines for RDD have not been established. Treatment plans are generally dependent on the extent and severity of the individual patient’s disease. Treatment guidelines have included operation, chemotherapy, steroids or other immunomodulatory medications. Reports have indicated a promising potential for the use of surgical intervention in the management of the cutaneous form of the RDD with no recurrence at distant follow-ups [7-8].

Our patient’s case was refractory to multiple excisions due to the metastatic-like behavior of this subset of Rosai-Dorfman lesions. Thus, chemotherapy with cladribine was necessary to effect a cure.

To our knowledge, we present the second case of recurrent cutaneous RDD and the first such disease with metastatic- and sacroma-like behavior. For similar cases, we recommend the early combined use of wide local excision and intravenous cladribine chemotherapy.

## Conclusions

Cutaneous RDD is an extremely rare and complex benign soft tissue disorder. We present the second case of recurrent cutaneous RDD and one with rare metastatic- and sarcoma-like behavior. Chemotherapy with intravenous cladribine in combination with wide local excisions led to regression of disease, and this combination may be the optimal treatment for such aggressive cutaneous RDD.
